# Parotid Metastases from Head–Neck Cutaneous Squamous Cell Carcinoma: A Prognostic Stratification

**DOI:** 10.3390/curroncol33070414

**Published:** 2026-07-10

**Authors:** Giulia Togo, Luca Calabrese, Giovanni dell’Aversana Orabona, Franco Ionna, Francesco Longo, Renato de Falco, Pietro Perotti, Ottavio Piccin, Luca Gazzini

**Affiliations:** 1 Maxillofacial and ENT Surgery Unit, Istituto Nazionale Tumori di Napoli IRCCS “G. Pascale”, 80131 Naples, Italy; 2 Department of Otolaryngology-Head and Neck Surgery, Hospital of Bolzano (SABES-ASDAA), Teaching Hospital of the Paracelsus Medical Private University (PMU), 39100 Bolzano, Italy; 3 Head and Neck Section, Department of Neurosciences, Reproductive and Odontostomatological Science, Federico II University of Naples, 80138 Naples, Italy; 4 Laboratory Medicine Unit, Istituto Nazionale Tumori, IRCCS Fondazione “G. Pascale”, 80131 Naples, Italy; 5 Department of Otorhinolaryngology-Head and Neck Surgery, “S. Chiara” Hospital, Azienda Provinciale per I Servizi Sanitari (APSS), 38123 Trento, Italy; 6 Centre for Medical Sciences-CSIMed, University of Trento, 38123 Trento, Italy

**Keywords:** parotid metastasis, cutaneous squamous cell carcinoma, staging stratification

## Abstract

Background/Objectives: Cutaneous squamous cell carcinomas (cSCC) of the head and neck district are among the most common non melanocytic malignant skin carcinomas. The proposal to differentiate, within the N stage, parotid metastases from lateral cervical metastases, originates from the different prognostic value of the metastatic region involved. Methods: We retrospectively evaluated 61 patients, surgically treated for parotid metastases from cSCC between January 2002 and June 2023, in four Departments of Surgery, to assess the geographic distribution of parotid metastases and to describe their recurrence patterns, to evaluate the prognostic value of the number of affected lateral cervical lymph nodes (LN) and the number of positive intra-glandular lymph nodes (IGLN) and to identify the main prognostic histopathological factors. Results: Our results did not show significant differences between participating centers in the distribution of parotid metastases, nor in their recurrence rates. However, our results highlight how adjuvant radiotherapy is deeply associated with the Overall Survival (OS), improving survival rates in patients with advanced-stage neoplasms (Odds Ratio 5.0), although causality cannot be inferred because of the retrospective study design. Moreover, a statistically significant correlation was found between the major inflammatory biomarkers and the OS. The presence of IGLN was identified as one of the main factors associated with recurrence and poor prognosis in patients with cSCC and in particular, in patients with N3b nodal stage. Conclusions: our findings suggest that both LN and IGLN could be used to propose an additional staging stratification for the N parameter, thereby guiding the treatment strategy and postoperative follow-up for patients with parotid metastases from cSCC of the head and neck district.

## Simple Summary

Cutaneous squamous cell carcinoma of the head and neck frequently metastasizes to regional lymph nodes. However, the relative prognostic impact of metastases involving the parotid gland compared with those affecting the lateral cervical lymphatic basin remains a matter of debate. In the present series, particular attention was paid to the distribution and burden of nodal disease, evaluating both intraglandular and lateral cervical lymph node involvement. The results suggest that the number of metastatic lateral cervical lymph nodes and the presence of intraglandular nodal metastases provide relevant prognostic information and may contribute to a more accurate stratification of patient risk. Moreover, adjuvant radiotherapy was associated with improved overall survival, with the greatest benefit observed in patients with more advanced disease, although the retrospective design of the study precludes conclusions regarding causality. Notably, intraglandular lymph node positivity emerged as a factor associated with a higher likelihood of recurrence and less favorable clinical outcomes. These results support incorporating both lateral cervical and intraglandular lymph node status into future nodal classification frameworks, potentially enhancing prognostic accuracy and informing postoperative management strategies in patients with parotid metastases from cutaneous squamous cell carcinoma.

## 1. Introduction

Cutaneous squamous cell carcinoma (cSCC) is one of the most common malignant neoplasms [1,2,3,4,5,6,7,8] and represents the second most frequent non-melanoma skin cancer, accounting for approximately 20–30% of cases. Its incidence ranges from 5 to 499 per 100,000 individuals, with marked geographical variability [2].

The development of cSCC is influenced by multiple environmental and patient-related factors. Among these, chronic exposure to ultraviolet radiation is considered the principal carcinogenic driver. UVB radiation exerts a stronger mutagenic effect than UVA radiation, although both wavelengths contribute to the accumulation of DNA damage and tumor development [4]. As a result, the incidence of cSCC is higher among fair-skinned individuals with a long history of sun exposure [9].

Approximately 80% of cSCCs arise in the head and neck region [10], reflecting greater cumulative solar exposure compared with other anatomical sites [3]. Established risk factors include chronic sun exposure, Caucasian ethnicity, male sex, advanced age, and immunosuppression. Increasing incidence rates have been associated with ozone layer depletion, higher numbers of organ transplant recipients, and aging populations [11].

Clinically, cSCC typically presents as erythematous papules, plaques, or ulcerated lesions located on sun-exposed areas such as the nose, ears, cheeks, lips, and temporoparietal scalp. Lesions may be asymptomatic or painful, while advanced tumors frequently ulcerate or bleed.

The 7th edition of the American Joint Committee on Cancer (AJCC) TNM classification introduced a staging system applicable to non-melanoma skin cancers as a whole [12]. In the subsequent 8th edition, cutaneous squamous cell carcinoma was addressed as a separate disease entity. The updated classification also incorporated extranodal extension (ENE) into the assessment of nodal disease, allowing a more accurate estimation of prognosis [13].

Alternative classification systems have been developed to better predict outcomes. The O’Brien classification differentiates parotid from lateral cervical nodal involvement [14], reflecting their distinct prognostic implications [15,16,17].

According to the National Comprehensive Cancer Network (NCCN), cSCC is categorized into low- and high-risk disease based on recurrence and metastatic potential. High-risk features include intraparotid or cervical nodal involvement (≥2 lymph nodes, nodal size > 3 cm, or ENE), primary tumor size > 5 cm (T3), or invasion of adjacent structures (T4), for which adjuvant radiotherapy is recommended [18].

Patterns of lymphatic dissemination in head and neck cSCC have been extensively investigated [7,8,9,10,11,12,13,14,19], particularly regarding spread to the parotid gland and lateral cervical lymph nodes. The most frequent metastatic sites include intraglandular lymph nodes (IGLN), the submandibular region, and ipsilateral lateral cervical lymph nodes. Reported metastasis rates range from 2.3% at 5-year follow-up to 5.2% beyond 5 years [20].

The parotid gland contains approximately 12–15 lymph nodes, predominantly within the superficial lobe, embedded in a dense lymphatic network [21]. These nodes, classified as intraparotid or intraglandular (IGLN) [22], primarily drain lymph from the scalp, posterior skull, ears, cheeks, and temporal regions [23]. Unlike mucosal squamous cell carcinomas, cSCC frequently extends into the parotid parenchyma through extensive lymphatic connections with ipsilateral cervical lymph node basins [23].

According to the updated DAHANCA/EORTC consensus guidelines, the parotid lymph nodes are classified as Level VIII in the modified Robbins classification, providing a standardized anatomical framework for communication among surgeons, radiologists, pathologists and radiation oncologists [24]. Furthermore, Civantos et al. [25] demonstrated that lymphatic drainage in oral cavity squamous cell carcinoma may be redirected toward unexpected lymphatic basins in approximately 13.6% of patients, whereas lymphatic drainage in cutaneous SCC is generally more predictable. These findings further support meticulous preoperative ultrasound mapping of the neck, including Level VIII, in patients with head and neck cutaneous SCC.

Five-year survival rates range from 30% to 60%, depending on disease stage [26,27,28]. Approximately 85% of metastases are locoregional, while distant spread most commonly involves the lungs, liver, brain, skin, and bones. Lymph node metastases typically occur within 2 years after treatment of the primary tumor [29].

Parotid involvement is observed in 60–82% of patients with metastatic cSCC, whereas isolated lateral cervical metastases without parotid disease occur in only 18–41% of cases [3]. Patients with parotid metastases show a 26% incidence of clinically evident cervical disease and a 35% rate of occult cervical metastasis [16]. Overall metastatic rates are approximately 5% but may exceed 20% in high-risk tumors, particularly those of the auricle and temporoparietal scalp [21,22,23,30,31,32,33].

Lateral cervical lymph node metastases represent a major adverse prognostic factor and are more frequent in patients with parotid involvement, lymphovascular invasion (LVI), and perineural invasion (PNI) [31,32,34,35]. Additional negative prognostic factors include tumor size, recurrence, prior radiotherapy, immunosuppression, extracapsular spread, positive surgical margins, and anatomical location, particularly the auricle [19,34].

Surgical resection with histologically negative margins remains the primary treatment for head and neck cSCC [36,37]. When metastatic spread to the parotid gland is present, surgery is generally combined with postoperative radiotherapy in patients with recognized risk factors [37,38,39]. Depending on the extent of disease, treatment may involve superficial, total, or extended parotidectomy, whereas radical parotidectomy with facial nerve resection is reserved for selected cases with advanced local involvement [40,41]. In patients presenting with both parotid and cervical nodal metastases, lateral neck dissection is widely accepted as the preferred surgical approach. By contrast, the optimal management of a clinically node-negative (cN0) neck remains debated [42,43,44,45,46]. Radiotherapy is also considered in cases of unresectable disease or recurrent tumors, usually including the parotid bed and cervical nodal levels I–V within the treatment field, with prescribed doses ranging from 60 to 70 Gy [47].

Given the lack of a universally accepted staging system specifically addressing the prognostic impact of parotid involvement in metastatic cutaneous SCC, we additionally explored the prognostic performance of the parotid carcinoma staging system.

Given the prognostic impact of parotid and cervical lymph node involvement the aims of this study were:

- To analyse the geographical differences (North Italy vs. South Italy) in the distribution and Overall Survival (OS) of parotid metastases from cSCC;
- To evaluate the prognostic significance of the number of involved lateral cervical and intraglandular lymph nodes in head and neck cSCC with parotid metastases;
- To identify the main anatomopathological prognostic factors.

## 2. Materials and Methods

This retrospective study has evaluated 61 patients surgically treated for parotid metastases from cSCC between January 2002 and June 2023 at the Maxillofacial Surgery Unit of the University “Federico II” of Naples, at the Otorhinolaryngology and Head and Neck Surgery Unit of “San Maurizio Hospital” in Bolzan, at the Otorhinolaryngology Unit of Trento Hospital and the Maxillofacial and ENT Surgery Unit of the IRCCS National Cancer Institute “Fondazione G. Pascale” of Naples. The cohort consisted of 5 females and 56 males, aged from 56 to 95 (mean age of 79.78 ± 7.59 SD).

Inclusion criteria were as follows:

- Patients undergoing surgery for parotid metastases from cSCC of the head and neck region;
- Histological diagnosis of cSCC of the head and neck;
- No prior surgical, radio-therapeutic, or chemotherapeutic treatment for the same neoplasm or any other malignancy;
- Absence of active infection, chronic inflammation, autoimmune diseases, or other malignancies at the time of admission;
- Minimum follow-up period of 12 months;
- Age > 18 years;
- Preoperative imaging performed (CT or MRI).

Exclusion criteria included:

- Administration of neoadjuvant chemotherapy;
- Primary squamous cell carcinoma of the parotid gland;
- Missing essential clinical data;
- Presence of distant metastases (M+);

All patients underwent clinical examination to identify palpable parotid and/or cervical masses. Suspicious lesions detected by imaging (CT/MRI) were further evaluated by fine-needle aspiration (FNA) or core biopsy to assess malignancy.

Contrast-enhanced CT or MRI was performed to confirm regional metastases and to assess lymph node size, number, and location. Additionally, PET/CT was used to exclude distant metastases and to plan potential adjuvant radiotherapy.

Clinical and pathological data were retrieved retrospectively from medical records. Information collected included demographic characteristics, primary tumor location, site of parotid involvement (superficial or deep lobe), administration of neoadjuvant therapies, occurrence of local or regional recurrence, type of surgical treatment performed, and pathological stage according to the UICC/AJCC 8th edition TNM classification. The number of metastatic intraparotid and cervical lymph nodes was documented for each patient. Additional pathological variables, including extranodal extension (ENE), perineural invasion (PNI), and lymphovascular invasion (LVI), were recorded. Preoperative laboratory parameters comprised platelet, neutrophil, and lymphocyte counts. From these values, the platelet-to-lymphocyte ratio (PLR), neutrophil-to-lymphocyte ratio (NLR), and systemic immune-inflammation index (SII) were calculated as follows:

- PLR = platelet count/lymphocyte count;
- NLR = neutrophil count/lymphocyte count;
- SII = (neutrophil count × platelet count)/lymphocyte count.

Surgical treatment of the primary cSCC involved excision with clear margins, performed prior to or concurrent with parotid metastasis surgery.

Parotid metastasis surgery consisted of:

- Superficial parotidectomy for metastases confined to the superficial lobe;
- Total conservative parotidectomy for metastases involving the deep lobe;
- Radical parotidectomy with sacrifice of one or more branches of the facial nerve in cases of nerve adherence;
- Extended parotidectomy with skin resection for cutaneous involvement or petrous bone drilling for infiltration of the external auditory canal.

All patients underwent ipsilateral lateral cervical lymph node dissection simultaneously with parotidectomy. The extent of neck dissection was determined by clinical and radiological findings:

- Elective neck dissection (END) for clinically and radiologically negative neck (cN0);
- Modified radical neck dissection (MRND) for clinically or radiologically evident lateral cervical metastases (cN+);

Postoperative radiotherapy was administered to patients with advanced AJCC stage (III/IV), positive surgical margins, perineural invasion, lymph node metastases, or lymphovascular invasion. All patients receiving postoperative radiotherapy were discussed within a Multidisciplinary Tumor Board and treated according to current international recommendations and NCCN guidelines regarding indications, treatment fields, timing, and radiation dose.

Follow-up data were obtained from outpatient post-surgical evaluations. Follow-up schedule included:

- Clinical examinations every 2 months during the first year, every 3 months during the second year, and every 6 months for the following 3 years;
- Neck ultrasonography every 6 months or as clinically indicated;
- Annual CT or MRI scans for the first 2 years.

The last follow-up was conducted in June 2024.

Postoperative radiotherapy was recommended according to the multidisciplinary evaluation and current institutional practice, mainly in the presence of high-risk pathological features such as extracapsular/extranodal extension, multiple metastatic lymph nodes, positive or close surgical margins, perineural invasion, lymphovascular invasion, or advanced nodal disease. Radiotherapy was generally initiated within 6–8 weeks after surgery, provided adequate wound healing had been achieved. Although treatment protocols varied slightly among the participating institutions and evolved during the study period, the prescribed dose generally ranged between 60 and 66 Gy, delivered in conventional daily fractions (1.8–2.0 Gy/fraction), with dose adaptation according to margin status, extent of disease, and individual patient characteristics.

Overall survival (OS) was calculated from the date of surgery to death from any cause or last follow-up, in addition, an exploratory subgroup analysis was performed in patients with N3b disease after excluding subjects who died from causes unrelated to cutaneous squamous cell carcinoma, with the aim of reducing the influence of competing causes of death and better evaluating disease-related oncologic outcomes.

Normality of continuous variables was assessed using the Kolmogorov–Smirnov test, and homogeneity of variances was evaluated using Levene’s test. The mean and standard deviation were used to describe continuous parametric variables, while the median and interquartile range (IQR) were reported for continuous non-parametric variables. Counts and percentages were used to summarize categorical variables.

Differences between continuous variables across groups were analyzed using Student’s *t*-test or the Mann–Whitney U test, depending on the distribution and variance of the variables.

Differences between categorical variables were assessed with Pearson’s Chi-square test, while Fisher’s exact test was applied for comparisons involving two dichotomous variables.

Survival curves for different groups were estimated using the Kaplan–Meier method, and differences between curves were evaluated using the log-rank test.

Associations between the variables of interest and the outcome variable (overall survival, OS) were assessed using binary logistic multivariable regression. Variables entered into the multivariable model were selected based on their clinical relevance and evidence of association with the outcome. A stepwise selection procedure based on Wald statistics was adopted with significance levels set at 0.05 and 0.10, respectively.

Receiver operating characteristic (ROC) curve analysis was performed, and the area under the curve (AUC) was calculated to assess sensitivity and specificity.

Missing data were assessed for all variables. Due to the retrospective nature of the study, analyses were conducted using a complete-case approach, and no data imputation was performed. For all tests, *p*-values < 0.05 were considered statistically significant. Statistical analyses were performed using SPSS software version 28.0 (SPSS Inc., Chicago, IL, USA).

## 3. Results

A total of 61 patients were enrolled in our study according to the inclusion and exclusion criteria described.

Patient staging was performed following the current AJCC staging system for cutaneous squamous cell carcinoma (cSCC). Fifteen patients were classified as T1, 17 as T2, 16 as T3, 2 as T4a, and in 11 patients the primary T category could not be determined, thus classified as Tx.

Regarding nodal involvement (N), no patients were classified as N0, consistent with the inclusion criterion requiring the presence of parotid metastases. Seventeen patients were N1, one patient N2a, nine patients N2b, one patient N2c, one patient N3a, and 32 patients were classified as N3b.

Additionally, we staged the tumors according to the parotid carcinoma staging system, despite this not being standard practice per international guidelines—except for squamous cell carcinomas that can be definitively considered primary parotid tumors—to evaluate whether this staging could better stratified the patient survival. Using this staging system, 12 patients were classified as T1, 11 as T2, 19 as T3, and 19 as T4a. For lateral cervical lymph node metastases, 32 patients were N0, 8 were N1, 10 were N2b, 1 was N2c, and 10 were N3b.

The main patient and tumor characteristics are summarized in Table 1.

Regarding the survival outcomes, a total of 37 patients (60.66%) were alive at the last follow-up, whereas 24 patients (39.34%) had died. Overall Survival (OS) rates were 88.9% at 1 year, 72.3% at 2 years, 60% at 3 years, and 41.2% at 5 years.

**Figure 1:**
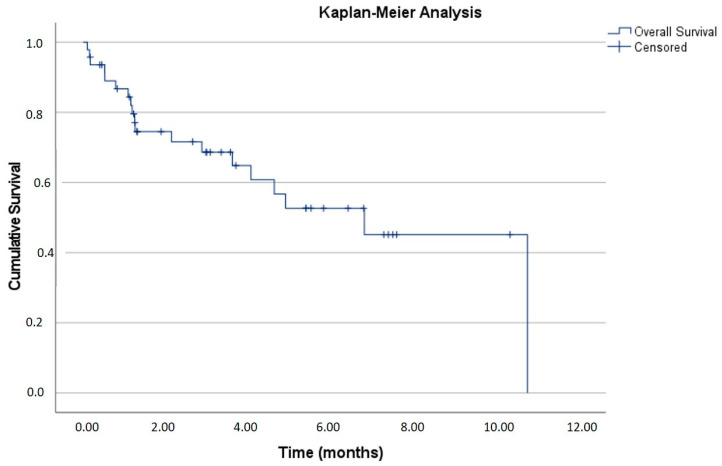
Kaplan–Meier curve for overall survival (OS) in the study cohort (*n* = 61).

Figure 1 illustrates the Kaplan–Meier survival curve for OS.

No statistically significant difference in OS was observed among the different centers (Pearson’s Chi-square test, *p* = 0.899). Further stratification grouping centers by geographic region (North Italy vs. South Italy) also did not reveal statistically significant differences (*p* = 0.903), suggesting that variations in cutaneous phototype, while potentially a risk factor for initial cutaneous squamous cell carcinoma (cSCC) development, do not retain prognostic value once parotid metastases have developed. Additionally, neither age (Mann–Whitney U test, *p* = 0.712) nor sex (Pearson’s Chi-square test, *p* = 0.975) were significant predictors of survival.

Concerning adjuvant therapy, 21 patients did not receive postoperative radiotherapy (RT); among them, 9 (42.86%) were alive at last follow-up and 12 (57.14%) had died. Conversely, 38 patients received adjuvant RT, with 28 (73.68%) alive and 10 (26.32%) deceased at last follow-up. Postoperative RT was significantly associated with improved OS (Pearson’s Chi-square test, *p* = 0.019; Fisher’s exact test, *p* = 0.026), underscoring the survival benefit of adjuvant radiotherapy in this cohort. Multivariate logistic regression analysis considering all factors significantly associated with OS (parotid pT stage, perineural invasion, adjuvant radiotherapy), age and sex yielded for postoperative RT an odds ratio (OR) of 5.0 (95%CI 1.370–18.320), indicating that patients undergoing adjuvant RT had a 5-fold increased likelihood of survival compared to those who did not. To further corroborate these findings and account for time-to-event dynamics, a Cox proportional hazards model using the same covariates was applied. Postoperative RT remained a significant prognostic factor (HR = 3.02, 95%CI 1.063–8.602).

**Figure 2:**
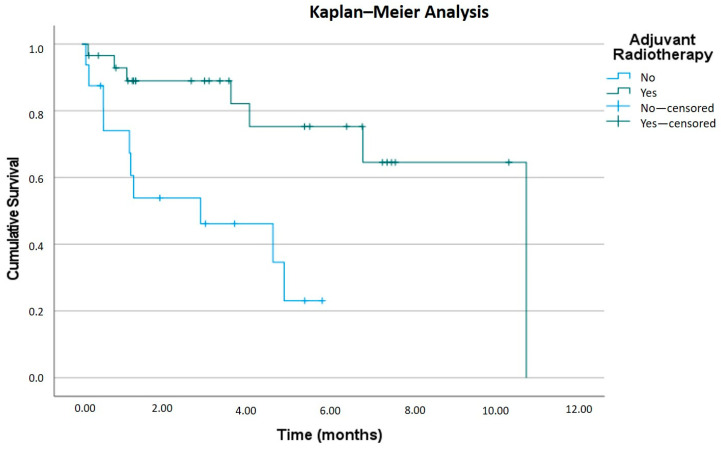
Kaplan–Meier estimates of overall survival in patients receiving (*n* = 38) or not receiving (*n* = 21) adjuvant radiotherapy.

Figure 2 displays Kaplan–Meier survival curves stratified by adjuvant RT status. OS was also analyzed in relation to key inflammatory biomarkers; neutrophil-to-lymphocyte ratio (NLR), platelet-to-lymphocyte ratio (PLR), and systemic immune-inflammation index (SII) showed no significant correlation with OS.

No statistically significant differences in OS were observed according to the primary cutaneous tumor T classification (Pearson’s Chi-square test, *p* = 0.261), including when T categories were grouped into early (T1–T2) versus advanced (T3–T4) stages (*p* = 0.404). Similarly, the primary tumor site (*p* = 0.508), histological grading (*p* = 0.375), lymphovascular invasion (LVI) (*p* = 0.453), and perineural invasion (PNI) (*p* = 0.146) were not significant prognostic factors for OS.

Conversely, applying parotid tumor staging (considering the parotid metastasis as a primary squamous carcinoma of the major salivary gland) showed a non-significant trend toward an association between parotid pT stage and OS (Pearson’s Chi-square test, *p* = 0.053). This association reached statistical significance when comparing early parotid stages (T1) versus advanced stages (T2–T4a) (Pearson’s Chi-square test, *p* = 0.014; Fisher’s exact test, *p* = 0.019), suggesting that a larger patient cohort might confirm this significance. Furthermore, histopathological features of the parotid lymph node metastases, such as PNI (*p* = 0.029), were significantly correlated with OS. If validated, these data imply that staging the parotid metastasis as a primary parotid tumor better stratifies risk than staging based on the primary cutaneous tumor.

Unexpectedly, the presence of intraparotid lymph node metastases (IGLN+) was not associated with prognosis (*p* = 0.219), nor was the number of positive intraglandular lymph nodes (*p* = 0.210).

The rationale for exploring this alternative classification stems from the fact that the current AJCC system groups together different patterns of regional disease spread, including parotid and cervical metastases, which may carry distinct prognostic implications.

Because ten patients classified as N3b died from causes unrelated to cutaneous squamous cell carcinoma during post-operative follow-up, an additional exploratory subgroup (32 patients) analysis was performed after excluding these patients, to refine the assessment of prognostic parameters. This analysis was conducted to reduce the impact of competing causes of death and to better assess disease-related oncologic outcomes within the N3b subgroup. The primary endpoint of the study remained overall survival, whereas this subgroup analysis was intended to provide supplementary information regarding disease-related outcomes in patients with advanced nodal disease.

In this subgroup of 32 patients, a statistically significant correlation between N3b stage and outcome (Pearson’s Chi-square test, *p* = 0.037) was demonstrated. Additionally, N3b stage correlated prognostically with inflammatory biomarkers: NLR (*p* = 0.048) and SII (*p* = 0.020).

**Figure 3:**
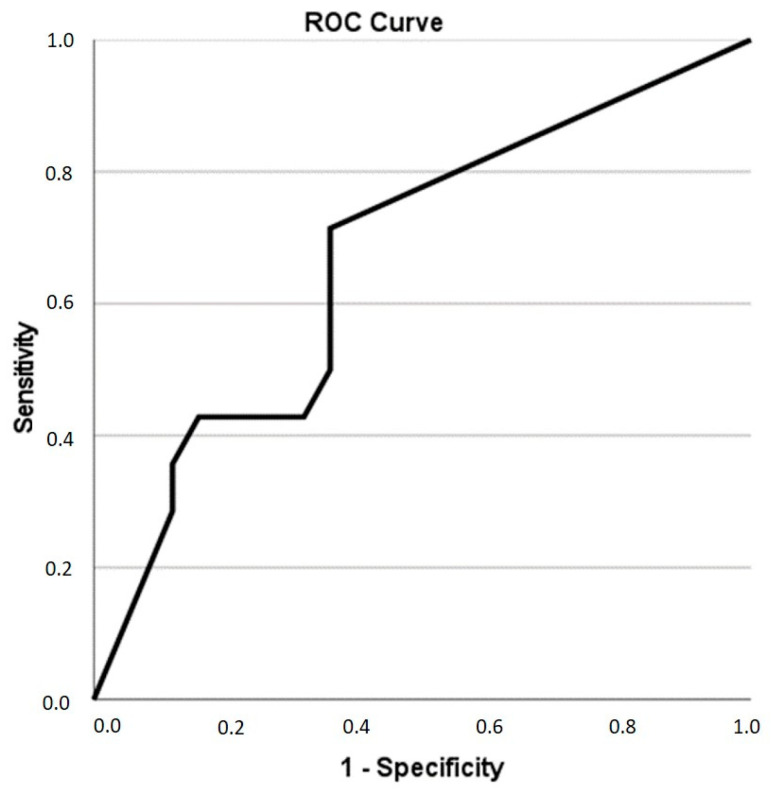
ROC Curve for IGLN ratio.

Notably, a strong correlation was found between N3b stage and positive intraglandular lymph nodes, which was significantly associated with outcomes (Pearson’s Chi-square test, *p* = 0.015; Fisher’s exact test, *p* = 0.033). The IGLN ratio (defined as positive IGLNs divided by total IGLNs) was evaluated by receiver operating characteristic (ROC) analysis. The ROC curve yielded an AUC of 0.669 (95% CI: 0.489–0.848). The optimal cutoff value, determined using the Youden index, was 0.0385, corresponding to a sensitivity of 71.4% and a specificity of 64.0%. Patients with an IGLN ratio >0.0385 tended to have poorer overall survival, suggesting a potential association between intraparotid lymph node involvement and unfavorable prognosis (Figure 3).

**Figure 4:**
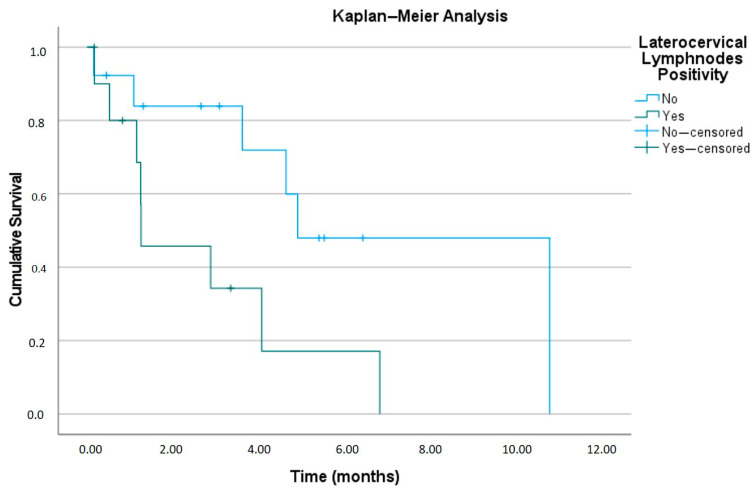
Kaplan–Meier estimates from the exploratory survival analysis performed in the N3b subgroup after exclusion of patients who died from causes unrelated to cutaneous squamous cell carcinoma, comparing patients with positive laterocervical lymph nodes (*n* = 16) to those without (*n* = 16).

In contrast, positive lateral cervical lymph nodes (LN+) in N3b patients showed a non-significant trend toward poorer OS (*p* = 0.053), confirmed by comparing outcomes between pN0 (LN−) and higher nodal stages (Kaplan–Meier–log rank *p* = 0.035) (Figure 4).

## 4. Discussion

Most cutaneous squamous cell carcinomas (cSCC) are located in sun-exposed areas of the head and neck district. Head and neck cSCCs are associated with a low rate of metastasis; specifically, they carry a 4–5% risk of metastasis to the parotid and cervical lymph nodes. Despite this low incidence, prognosis remains poor, with a 5-year overall survival (OS) rate ranging from 30% to 60%, regardless of stage [1].

Beyond directly impacting mortality in patients with head and neck cSCC, the presence of regional metastases significantly affects morbidity, as these patients often require multimodal treatment, including neck dissection, various extents of parotidectomy (with or without facial nerve preservation), and postoperative radiotherapy [31].

Diagnosis of these lesions is frequently delayed and affected patients tend to be elderly with multiple comorbidities. Parotid metastases necessitate surgical management consisting of parotidectomy combined with a lateral neck lymphadenectomy.

Consistent with the literature, the majority of patients are male [14]. The mean age of patients with parotid metastases from head and neck cSCC also aligns with previously published data, confirming that elderly patients are particularly susceptible to parotid metastases from cSCC [14].

The most common primary cSCC sites within the head and neck region include the auricle, temporal region, and forehead [42,43]. As described by Creighton et al. [43], the highest rates of parotid metastasis occur in cSCCs of the frontal region (85%), preauricular area (76%), and scalp (30%). Our findings corroborate those reported by Hirshoren et al. [43], which identified the scalp and auricle as the most frequent facial sites associated with cSCC metastases.

Despite multimodal therapeutic strategies, the 5-year OS of patients with parotid metastases from cSCC remains low, in agreement with literature data [14,28,44], and appears strongly correlated with advanced tumor stage.

Among potential diagnostic and therapeutic strategies to improve OS in this patient population, early diagnosis and appropriate staging are paramount.

Besides cross-sectional imaging, high-resolution ultrasound plays a fundamental role in the preoperative evaluation of superficial cervical and intraparotid lymph nodes. In particular, real-time ultrasound-guided fine-needle aspiration cytology (US-guided FNAC) allows accurate cytological confirmation of suspicious metastatic lymph nodes, improving diagnostic accuracy and supporting multidisciplinary treatment planning. Furthermore, the recently proposed Lymph Node Reporting and Data System (LN-RADS) provides a standardized ultrasound classification of superficial lymph nodes that may improve communication between radiologists and clinicians and facilitate patient stratification [48].

Deihles et al. [49] demonstrated that 37% of patients with parotid metastases from cSCC received a correct diagnosis only at an advanced disease stage, and 62% had incomplete histological diagnosis.

Increasing the radicality of the surgical approach may represent an effective strategy to improve OS, as supported by the use of more extensive surgery [45]. The advent of immunotherapy, in addition to conventional surgical and radiotherapeutic treatment, may further enhance OS in patients with parotid metastases from cSCC [50].

Several prognostic factors influencing OS in patients with parotid metastases from head and neck cSCC have been identified, including:

- Extranodal extension (ENE) [12,13,14,15,16,17,19,46,51,52,53,54];
- Lymph node ratio (LNR), defined as the ratio of positive lymph nodes to the total number of lymph nodes removed during lateral neck dissection [55];
- Involvement of intraparotid/intraglandular lymph nodes [18,56];
- Histological grade of the primary tumor [57];
- Immunosuppression [58];
- Margins of the primary tumor [59,60].

Numerous classification systems integrating these prognostic factors exist in the literature, consistent with our results [12,13,14,61].

Staging systems are critical tools for prognostic stratification and guide clinicians in treatment planning according to tumor risk. Currently, the AJCC 8th edition [13] represents the reference guideline for staging patients with various cancers, including cSCC. These guidelines are continuously updated to incorporate important prognostic features. Since its first publication in 1997, eight editions have been released. The 7th edition [12] first incorporated key features such as depth of invasion, perineural invasion, and histologic grade.

Published in 2017, the 8th edition introduced several significant risk factors based on cohort studies from the 7th edition, including perineural invasion, bone erosion, subcutaneous tissue invasion, and depth of invasion (DOI) > 6 mm. Regarding nodal metastases (N category), extranodal extension (ENE) is now considered, indicating a more advanced stage.

Nevertheless, controversy persists regarding the accuracy of this staging system for prognostic stratification in head and neck cSCC patients.

The rationale for exploring this alternative classification stems from the fact that the current AJCC system groups together different patterns of regional disease spread, including parotid and cervical metastases, which may carry distinct prognostic implications, as reported by Hirshoren et al., auricular cSCC is associated with poor prognosis [42]; however, in the AJCC 8th edition, primary tumor characteristics for lesions classified as T1–T2 do not impact staging.

The poor prognosis of advanced lesions with parotid metastases is widely discussed in the literature [57,62,63]. Our study demonstrated no statistically significant difference in OS according to the primary cutaneous T classification (Pearson’s chi-square *p* = 0.311), a finding further confirmed when grouping early T (T1–T2) versus advanced T (T3–T4) tumors (Pearson’s chi-square *p* = 0.850).

These findings may be attributable to the limited sample size, which could hinder achieving statistical significance, or may reflect that once parotid nodal metastases occur, prognosis is driven primarily by the metastatic disease rather than the characteristics of the primary cutaneous tumor.

Conversely, when stratifying prognosis based on parotid tumor staging—considering the parotid metastasis as a primary squamous cell carcinoma of the parotid gland—the difference between cT stages reached statistical significance.

This finding may be due to the limited sample size preventing statistical significance, or it may indicate that once parotid nodal metastases occur, prognosis is no longer influenced by the extent or histopathological features of the primary cutaneous tumor but solely by the parotid metastasis itself.

If confirmed and supported by a larger series and further scientific evidence, these data may suggest that the staging system that best stratifies risk in these patients is not that of the primary cutaneous tumor but rather the staging of the parotid nodal disease. This is further supported by the correlation identified between prognosis and histological factors of the parotid metastasis, unlike histological factors of the primary cutaneous tumor, which did not correlate with OS.

Recently, Moeckelmann et al. [64] evaluated the accuracy of the AJCC 8th edition nodal staging system in a retrospective Australian cohort of 382 head and neck cSCC cases. The current nodal staging system failed to provide risk stratification, suggesting that cSCC may require an independent nodal staging system.

In 2002, O’Brien et al. [14] published the first report demonstrating better prognosis for parotid involvement compared to cervical involvement, hypothesizing that prognostic discrimination might be improved by separating parotid from cervical disease.

Ch’ng et al. [16] further explored this, showing worse prognosis associated with progression of parotid or cervical disease and the impact of combined parotid and cervical metastases versus single-site metastasis on outcomes.

Our study confirms these findings, showing that intraparotid lymph node positivity predicts recurrence, and in N3b-stage tumors, intraparotid lymph node involvement predicts poorer outcomes.

Since then, various studies have yielded heterogeneous results [13,14,15,16,45,53,54,65,66].

Conversely, a study by Hirshoren et al. [42] involving 183 head and neck cSCC metastasis cases found no association between OS and metastasis site (parotid vs. neck vs. both), identifying only an association with total lymph node ratio. These results align with other studies on different head and neck cancers [67].

Our data did not show a statistically significant correlation between positive lateral cervical lymph nodes and OS in N3b patients, likely due to the limited sample size. However, the observed trend suggests that the presence of laterocervical nodal involvement may be associated with poorer oncologic outcomes and warrants further investigation in larger cohorts.

The results obtained in the N3b subgroup should be interpreted cautiously. Given the limited sample size and the exclusion of patients who died from causes unrelated to cutaneous squamous cell carcinoma, these analyses were not intended to replace the primary overall survival analysis but rather to provide complementary information on disease-related outcomes in patients with advanced nodal disease. These findings are complementary to, rather than a substitute for, the overall survival analyses conducted in the entire cohort.

Although preliminary data suggest cervical metastases may be associated with poorer prognosis compared to parotid metastases, there is insufficient evidence in the literature to support this distinction.

From an anatomical perspective, the recognition of intraparotid lymph nodes as Level VIII according to the modified Robbins classification may further contribute to standardized reporting of nodal disease, improve multidisciplinary communication, and optimize surgical and radiotherapeutic planning in patients with advanced head and neck cutaneous SCC [24].

The prognostic importance of total lymph node ratio on OS has been previously described for patients with mucosal head and neck cSCC nodal metastases [19]. Total lymph node ratio correlates with OS but not with locoregional disease control.

It is challenging to explain why lymph node ratio correlates with OS but not locoregional control. It is unlikely related to the extent of surgery, reflected by the total number of nodes removed (denominator), which also does not correlate with locoregional control. However, it may relate to the number of positive nodes (numerator), which correlates with OS but not locoregional control.

Girardi et al. [68] highlighted the prognostic value of metastasis site in head and neck cSCC. They demonstrated that primary tumor site, tumor diameter, DOI, deep invasion into subcutaneous fat, presence of metastases at diagnosis, stage, and number of positive nodes all correlated with disease-free survival (DFS) in their cohort.

Regarding management of the primary tumor and parotid metastases in cSCC patients, there is general consensus that surgery followed by adjuvant radiotherapy, when indicated, is the most appropriate treatment strategy [40,41,42,47,69,70,71,72,73,74,75].

Accurate preoperative ultrasound assessment of Level VIII lymph nodes combined with US-guided FNAC may further optimize patient selection for surgery and improve pathological staging [48].

Consistent with the literature, our results confirm that receiving postoperative adjuvant radiotherapy is significantly associated with improved OS (Pearson’s chi-square *p* = 0.011). This finding is especially noteworthy considering that adjuvant radiotherapy is typically recommended for patients with advanced-stage disease or unfavorable histopathological features, who would generally have a reduced OS. Our data demonstrate that adjuvant radiotherapy actually tend to reverses this trend, with patients receiving it having an 5-fold higher likelihood of survival compared to those who did not and so the administration of RT appears to reverse this trend. However, these findings should be interpreted cautiously given the retrospective nature of the study and the non-randomized allocation of adjuvant treatment. Patients receiving postoperative radiotherapy demonstrated more favorable survival outcomes despite the presence of features traditionally associated with poorer prognosis. Nevertheless, this observed association should not be interpreted as evidence of a causal effect.

This study is limited by its retrospective design and small sample size, which may reduce statistical power and increase susceptibility to confounding. The multicenter inclusion over a long time span, could potentially have caused the non-availability of some patient’s specific informations and may have led to an heterogeneity related to evolving imaging techniques, pathological reporting, surgical approaches, and adjuvant treatment protocols. The multicenter design and the long study period inevitably resulted in some variability in postoperative radiotherapy indications, treatment timing, and dose prescription among participating institutions. Although these differences reflect real-world clinical practice, they may have contributed to treatment heterogeneity and should be considered when interpreting the association observed between adjuvant radiotherapy and survival outcomes.

Furthermore, prospective studies with larger cohorts and standardized protocols are warranted to get rid of any possible influence selection bias and unmeasured confounding. Moreover the therapeutic landscape of advanced cSCC has rapidly evolved with the introduction of immune checkpoint inhibitors. Although systemic treatment was not the focus of the present study, immunotherapy is increasingly becoming an integral component of the management of advanced and recurrent disease and may further influence future prognostic stratification models.

Although lateral neck dissection is the guideline-recommended approach for clinically positive neck (cN+) cSCC, management of the clinically negative neck (cN0) remains controversial, as elective surgery and irradiation yield comparable outcomes [40].

Dür et al. [76] reported that the incidence of occult metastases in head and neck cSCC exceeds the conventional threshold used for mucosal squamous cell carcinomas in the head and neck. Parotid lymph node status may be predictive of occult cervical nodal metastases, suggesting that elective lateral neck dissection or irradiation may not be necessary in patients without clinical or radiological evidence of parotid or cervical lymph node metastases.

O’Brien et al. [22] demonstrated that patients with intraparotid lymph node metastases have a high incidence of clinically evident (26%) and occult (35%) lateral cervical metastases. In line with other head and neck carcinomas, the presence of lateral cervical lymph node metastases correlates with decreased 5-year survival. Patients with parotid metastases but no cervical involvement have a 5-year overall survival rate of 65–70%, which drops to 30% with the development of multiple lateral cervical lymph node metastases (pN2).

Recent evidence has further emphasized the importance of tailored management strategies in advanced cSCC, particularly regarding elective neck treatment and the integration of systemic therapies and immunotherapy in selected high-risk patients [34,47,71].

## 5. Conclusions

In this multicenter retrospective cohort, postoperative radiotherapy was associated with improved OS. However, given the retrospective design and the potential influence of residual confounding and selection bias, these findings should be interpreted cautiously and require confirmation in larger prospective studies.

The presence of IGLN was identified as a major prognostic factor associated with recurrence risk and poorer prognosis in the subgroup of patients exhibiting nodal stage N3b. Moreover, our findings, if confirmed in larger cohorts, may suggest that both metastatic LN and positive IGLNs could be utilized to propose a refined stratification of the nodal (N) staging parameter. This enhanced stratification, if obtainable through additional analysis on a larger case study, would facilitate a more precise treatment planning and postoperative follow-up for surgically treated patients with parotid metastases from head and neck cSCC.

## Figures and Tables

**Table 1:** Main characteristics of the population.

	TOTAL (*n* = 61)	ALIVE (*n* = 37)	DECEASED (*n* = 24)
**SEX**			
M	56 (91.80%)	34 (91.89%)	22 (39.28%)
F	5 (8.20%)	3 (8.11%)	2 (40.00%)
**TUMOR (T) LOCALIZATION**			
Ear	22 (36.07%)	11 (29.73%)	11 (45.83%)
Temple	13 (21.31%)	8 (21.62%)	5 (20.83%)
Scalp	10 (16.39%)	6 (16.22%)	4 (16.67%)
Lip	1 (1.64%)	1 (2.70%)	0
Cheek	8 (13.11%)	7 (18.92%)	1 (4.17%)
Eyelid	4 (6.56%)	2 (5.41%)	2 (8.33%)
Unknown	3 (4.92%)	2 (5.41%)	1 (4.17%)
**SKIN STAGING**			
pT			
1	15 (24.59%)	12 (32.43%)	3 (12.5%)
2	17 (27.87%)	8 (21.62%)	9 (37.5%)
3	16 (26.23%)	9 (24,32%)	7 (29.17%)
4a	2 (3.28%)	2 (5.41%)	0
4b	0	0	0
Tx	11 (18.03%)	6 (16.22%)	5 (20.83%)
pN			
1	17 (27.87%)	11 (29.73%)	6 (25.00%)
2a	1 (1.64%)	0	1 (4.17%)
2b	9 (14.75%)	7 (18.92%)	2 (8.33%)
2c	1 (1.64%)	1 (2.70%)	0
3a	1 (1.64%)	1 (2.70%)	0
3b	32 (52.46%)	17 (45.95%)	15 (62.5%)
GRADING			
G1	1 (1.64%)	0	1 (4.16%)
G2	18 (29.51%)	10 (27.02%)	8 (33.33%)
G3	26 (42.62%)	17 (45.95%)	9 (37.50%)
Missing	16 (26.23%)	10 (27.03%)	6 (25%)
PERINEURAL INVASION (PNI)			
Yes	32 (53.33%)	17 (53.13%)	15 (46.87%)
No	28 (46.67%)	20 (71.43%)	8 (28.57%)
LYMPHOVASCULAR INVASION (LVI)			
Yes	20 (32.79%)	11 (29.73%)	9 (37.50%)
No	40 (65.57%)	26 (70.27%)	14 (58.33%)
Missing	1 (1.64%)	0	1 (4.17%)
MARGINS			
R0	39 (63.93%)	24 (64.86%)	15 (62.50%)
R+	17 (27.87%)	10 (27.03%)	7 (29.17%)
Missing	5 (8.20%)	3 (8.11%)	2 (8.33%)
**PAROTID STAGING**			
pT			
1	12 (19.67%)	11 (29.73%)	1 (4.17%)
2	11 (18.03%)	4 (10.81%)	7 (29.17%)
3	19 (31.15%)	11 (29.73%)	8 (33.33%)
4a	19 (31.15%)	11 (29.73%)	8 (33.33%)
pN			
0	32 (52.46%)	21 (56.76%)	11 (45.83%)
1	8 (13.11%)	5 (13.51%)	3 (12.50%)
2b	10 (16.39%)	5 (13.51%)	5 (20.83%)
2c	1 (1.64%)	1 (2.70%)	0
3b	10 (16.39%)	5 (13.51%)	5 (20.83%)
LINFOVASCULAR INVASION (LVI)			
Yes	16 (26.23%)	8 (21.62%)	8 (33.33%)
No	39 (63.94%)	27 (72.97%)	12 (50.00%)
Missing	6 (9.84%)	2 (5.41%)	4 (16.67%)
PERINEURAL INVASION (PNI)			
Yes	30 (49.18%)	15 (40.54%)	15 (62.5%)
No	27 (44.26%)	20 (54.05%)	7 (29.17%)
Missing	4 (6.56%)	2 (5.41%)	2 (8.33%)
MARGINS			
R0	31 (50.82%)	21 (56.76%)	10 (41.67%)
R1	13 (21.31%)	6 (16.22%)	7 (29.17%)
R CLOSE	7 (11.48%)	4 (10.81%)	3 (12.50%)
Missing	10 (16.39%)	6 (16.22%)	4 (16.67%)
INTRAGLANDULAR LYMPH NODES (IGLN)			
Yes	19 (31.15%)	9 (24.32%)	10 (41.66%)
No	34 (55.74%)	22 (59.46%)	12 (50.00%)
Missing	8 (13.11%)	6 (16.22%)	2 (8.33%)
**ADJUVANT RADIOTHERAPY**			
Yes	38 (62.30%)	28 (75.68%)	10 (41.66%)
No	21 (34.43%)	9 (24.32%)	12 (50.00%)
Missing	2 (3.28%)	0	2 (8.33%)
**IPSILATERAL CERVICAL LYMPH NODES (LN)**			
Yes	21 (34.43%)	10 (27.03%)	11 (45.83%)
No	32 (52.46%)	21 (56.76%)	11 (45.83%)
Missing	5 (8.20%)	3 (8.11%)	2 (8.33%)

## Data Availability

The original data presented in the study are openly available in Zenodo at https://doi.org/10.5281/zenodo.17455973 (accessed on 27 October 2025).
